# Interspecific hybridization in *Cucumis* leads to the divergence of phenotypes in response to low light and extended photoperiods

**DOI:** 10.3389/fpls.2015.00802

**Published:** 2015-10-02

**Authors:** Xiaqing Yu, Benita Hyldgaard, Eva Rosenqvist, Carl-Otto Ottosen, Jinfeng Chen

**Affiliations:** ^1^State Key Laboratory for Crop Genetics and Germplasm Enhancement, College of Horticulture, Nanjing Agricultural UniversityNanjing, China; ^2^Department of Food Science, Aarhus UniversityÅrslev, Denmark; ^3^Department of Plant and Environmental Sciences, University of CopenhagenTaastrup, Denmark

**Keywords:** allotetraploid, chlorophyll, *Cucumis*, low light, photoperiod, photosynthesis

## Abstract

With the aim of improving shade tolerance of cucumber, *Cucumis* × *hytivus*, a newly synthesized allotetraploid, was obtained by crossing a shade tolerant wild relative, *Cucumis hystrix*, with a cultivated cucumber, *Cucumis sativus* L. ‘BejingJietou.’ The results show that the new *C. × hytivus* only partly is an intermediate hybrid and it has not only chlorophyll deficiency, which recovers during leaf development, but also lower carotenoid content. Three light conditions with the combination of different light intensities and photoperiods were employed to investigate the photosynthetic response of these three *Cucumis* species to low light and long photoperiod. The consistent order of *P*_max_ and DW_S_ being lowest in *C. hystrix*, medium in *C.* × *hytivus* and highest in ‘BejingJietou’ suggests the three species to have genetically different photosynthetic efficiency, which relates well with the natural habitats of the parent species and the hybrid as intermediate. *C.* × *hytivus* appears to be inhibited by the low light levels to the same extent as the cultivated ‘BeijingJietou,’ which indicates neither improvement of shade tolerance nor hypothetical heterosis effect in *C.* × *hytivus*. However, unexpectedly, the PSII of *C. hystrix* was affected by the long photoperiod in the long term, suggested by the decrease of *F*_v_/*F*_m_. This sensitivity toward day length has not been passed on to *C.* × *hytivus*.

## Introduction

Plants have evolved a number of mechanisms to acclimate to changing light levels, for example by changing the size of the light-harvesting complexes (LHCs) and leaf thickness to enable efficient capture and use of light (Lichtenthaler et al., 1981; Walters and Horton, 1994). Cucumber (*Cucumis sativus* L., 2n = 14) is one of the most important vegetable crops in many countries and more than 75% (54.3 million tons) were produced in China (FAOSTAT 2013, data available at http://faostat.fao.org/). During the winter in China, low irradiance is the major limiting factor for cucumber growth and yield in the protected production (Ma et al., 1998). The improvement of shade-tolerance in cucumber has been slow due to the narrow genetic base of cucumber (3–12% polymorphism; Knerr et al., 1989; Dijkhuizen et al., 1996).

An interspecific cross was made in *Cucumis* with the shade adapted *Cucumis hystrix* Chakr. and a cultivated cucumber, *C*. *sativus* L. ‘BejingJietou’ (hereafter referred to as ‘BeijingJietou’) as parent species (Chen et al., 1994). *C. hystrix* is a wild species, exhibiting dark green leaf color, originating from the understory of isolated rainforests in Xishaungbanna, Southern China (Chen et al., 1994). Due to the close relationship with the cultivated cucumber, it was considered of great importance as it could possibly contribute to broaden the narrow genetic base of cucumber through interspecific hybridization. *Cucumis* × *hytivus* J.-F. Chen and J. H. Kirkbr. (2n = 38) is a fully fertile allotetraploid obtained through a successful cross between *C. hystrix* (2n = 24) and ‘BejingJietou’ (2n = 14; Chen and Kirkbride, 2000). When a successful cross is made between two species, the phenotype of the new hybrid may not be an intermediate between the parental species. In some cases hybrid plants or animals grow more vigorously than their parent species, known as the phenomenon of heterosis or hybrid vigor (Birchler et al., 2010). This effect may also be observed in allopolyploids (Baranwal et al., 2012; Chen, 2013). *C.* × *hytivus* is a not only a hybrid, but also an allotetraploid (2n = 38), which makes it an interesting model to study the interspecific hybridization effect. Polyploidy is a fundamental complex biological mechanism and polyploids have been shown to be able to cope better, such as being more invasive, than their diploid progenitors in harsh environment (te Beest et al., 2012).

A preliminary study of *C.* × *hytivus* showed indications of low light tolerance such as low light compensation point (LCP; Qian et al., 2002). However, *C*. × *hytivus* possess a yellow–green color of the youngest leaves. This unique characteristic of yellow–green young leaves is expressed in the subsequent progenies indicating that the trait is stable in inheritance rather than being an accidental incident in one generation. Furthermore, we have observed that the yellow–green young leaves of *C.* × *hytivus* can recover to ‘normal’ green under the treatment of long photoperiod (personal observation). Longer day length with unchanged daily light integral (DLI) has been shown to result in a higher concentration of chlorophyll (hereafter Chl) in tomato (van den Boogaard et al., 2001). Therefore, it leaves the question if *C.* × *hytivus* in a similar fashion increase the content of Chl when exposed to longer day length. Moreover, the details of how the newly synthesized species *C.* × *hytivus* and its parents differ in their photosynthetic response to longer photoperiod and low light and whether the shade-tolerance had been passed on from the wild shade-adapted parent to the hybrid needed to be explored.

The objective of this study was to examine the pigment composition of the three species under different light intensities and photoperiods and to investigate whether *C.* × *hytivus* shows heterosis and thereby would allow the species to improve its acclimatization of photosynthesis to low light, low DLI and long photoperiod compared to its diploid parents.

## Materials and Methods

### Plant Material

Three species of *Cucumis* were used: the wild species *C. hystrix* Chakr. (2n = 2x = 24, genome HH), the synthesized species *C.* × *hytivus* J.-F. Chen and J. H. Kirkbr. (2n = 4x = 38, genome HHCC) and the cultivated cucumber *C. sativus* L. ‘BeijingJietou’ (2n = 14, genome CC). The seeds were sown at the end of March 2012 and grown under controlled greenhouse conditions (26/20°C day/night, ambient CO_2_, RH 60–70% and 20 h photoperiod with a combination of natural and supplemental light (SON-T 400W, Philips, Eindhoven, The Netherlands, red/far-red ratio: 1.2 Shibuya et al., 2012). Since the three species had different growth rates, they were cultivated in the greenhouse for 2 months to produce cuttings of equal size. The cuttings were planted in plastic pots (11-cm diameter, 0.5 L) filled with a peat based potting mix (Pindstrup 2, Pindstrup Mosebrug A/S, Ryomgaard, Denmark) and irrigated and fertilized regularly with a nutrient solution with N:P:K of 160:35:190, pH of 5.8, electric conductivity of 1.8.

### Light Treatments

Prior to the light treatments, the plants were grown in the greenhouse from June 4, 2012 until June 25. In the experiment, three light treatments were chosen to simulate different winter light conditions in northern China. The three treatments were created by combining 2 days-lengths and two different DLI, by a combination of lamps and shade screens. The light treatments were (1) low light with short day: 14 h/10 h light/dark with a mean value of 70 μmol m^-2^ s^-1^ (LL/SD, DLI: 3.5 mol m^-2^ day^-1^), (2) intermediate light with long day: 22 h/2 h light/dark with a mean value of 90 μmol m^-2^ s^-1^ (IL/LD, DLI: 7.1 mol m^-2^ day^-1^) and (3) high light with short day: 14 h/10 h light/dark with mean value of 140 μmol m^-2^ s^-1^ (HL/SD, DLI: 7 mol m^-2^ day^-1^). The experiment was conducted in a greenhouse compartment in a glasshouse (Aarhus University, Årslev, Denmark) fully covered by shade screens to reduce the natural light. Climate was controlled to 26/20°C day/night, ambient CO_2_, RH 60–70% in all treatments. A LI-190 Quantum Sensor (Li-Cor, Lincoln, NE, USA) placed at plant height was used to monitor the photosynthetic photon flux densities (PPFDs). Lamps and shade screens were adjusted to ensure that the PPFD was close to the set points. The plants were placed randomized on the benches of the glasshouse and grown under each light treatment for a period of 21 days. There were no repetitions of light treatments.

### Chlorophyll Fluorescence and Gas Exchange Measurements

For chlorophyll fluorescence and gas exchange measurements, the first fully developed leaf from three randomly selected plants was used. Border plants were excluded. The chlorophyll fluorescence was measured using a MINI-PAM (Walz, Effeltrich, Germany). The maximum quantum efficiency of photosystem II, *F*_v_/*F*_m_ = (*F*_m_*-F*_o_)/*F*_m_ (where *F*_o_ is the minimal and *F*_m_ the maximal fluorescence yield in a dark-adapted leaf) was measured at 10:00 h each day with different intervals of three species. The leaves were dark-adapted for 30 min in dark leaf clips DLC-8 (Walz, Effeltrich, Germany; Baker and Rosenqvist, 2004). The photochemical efficiency of photosystem II, Φ_PSII_ = *F*_q_′*/F*_m_′ = (*F*_m_′*-F*_s_)/*F*_m_′ (where *F*_q_′ is the quenched fluorescence yield, *F*_m_′ is the maximal fluorescence during a saturating light pulse and *F_s_* is the steady-state fluorescence at any level of actinic PPFD) was measured at 11 h on 1, 8, and 18 days of treatments. The electron transport rate (ETR) is calculated by multiplying Φ_PSII_ × incident PPFD × 0.5 (two photons are used for exciting one electron, assuming equal distribution of excitation between photosystems II and I), and × 0.84 (Krall and Edwards, 1992). It was measured on the middle part of the leaf in a leaf clip holder (2030-B, Walz, Effeltrich, Germany) with an external halogen lamp (2050-HB, Walz, Effeltrich, Germany) that produced PPFD levels similar to each experimental treatment. Twenty saturating light pulses (during 10 min) were applied in actinic light to ensure steady-state values before measurement recordings.

An IRGA system (CIRAS-2; PP-systems, Amesbury, MA, USA) was used for gas exchange measurements using the same part of the leaf used for the chlorophyll fluorescence measurements with a leaf cuvette of 2.5 cm^2^ [leaf area (LA)] with a LED light unit. The CO_2_ level was set to 300 ppm, leaf temperature to 26°C and the cuvette flow was 200 cm^3^ min^-1^. To prevent photoinhibition, the light response measurements were initiated at 300 μmol m^-2^ s^-1^ going to zero light in steps and returning to the initial light level and continuing in steps up to 1400 μmol m^-2^ s^-1^ with nine light levels in total. After changing the light intensity, the plants were allowed to acclimate for at least 5 min before steady-state gas exchange were reached and logged. Measurements from the different species and treatments were made over a 2-days period on each occasion. No significant difference was found between measuring days (data not shown), therefore the data were pooled. Curve fits were made using Photosynthesis Work Bench (Li-Cor, Lincoln, NE, USA) to obtain the gas exchange parameters, including the LCP, light saturation point (LSP), maximum net photosynthesis rate (*P*_max_), and dark respiration (*R*_d_).

### Pigment Content

The development in Chl content was monitored non-invasively by a Dualex 4 (FORCE-A, Orsay, France) during the light treatment period (Cerovic et al., 2012). The Dualex 4 delivers non-destructive readings in units of μg cm^-2^ for the Chl content during the experiment (Cerovic et al., 2012). For each species, three random plants from each treatment were measured. Measurements were performed on the uppermost fully developed leaf of the plants and were taken on the same leaf at a 2 or 3-days interval (for *C.* × *hytivus*, initially more frequently). Readings were taken from three sections of both sides of the leaves and the mean value of each leaf was calculated. The pigment content [dry weight (DW) basis] of the same leaf was determined destructively after the experiment. Leaf samples were frozen in liquid nitrogen and stored in -80°C for later analysis. Pigments were extracted from plant tissue in cold 96% ethanol and the concentrations of the pigments were quantified by light spectroscopy (UV-VIS spectrophotometer, Shimadzu, Kyoto, Japan), light absorbance at 470, 648 and 664 nm according to Lichtenthaler (1987), including chlorophyll *a* (Chl *a*), chlorophyll *b* (Chl *b*), and total carotenoids (xanthophylls and carotenes, hereafter Caro).

### Plant Growth

Plant growth was determined as total LA, leaf dry weight (DW_L_) and shoots dry weight (DW_S_) per plant after the experiment. The LA was measured using a LI-3100C area meter (Li-Cor, Lincoln, NE, USA) and the leaves and stems were dried at 80°C for 24 h for DW determination. Specific leaf area (SLA) was calculated as SLA = LA/DW_L_.

### Data Analysis

A two-way analysis of variance (ANOVA) was performed to reveal the differences between the species and treatments within 1 day of measurements. The software R (i3862.15.0, www.r-project.org/) was used for the statistical analysis. Mean separations was done using the Duncan Multiple Range Test of *P* < 0.05. SPSS 16.0 (SPSS, Inc., Chicago, IL, USA) was used for the Pearson correlation analysis.

## Results

### Morphology

**FIGURE 1:**
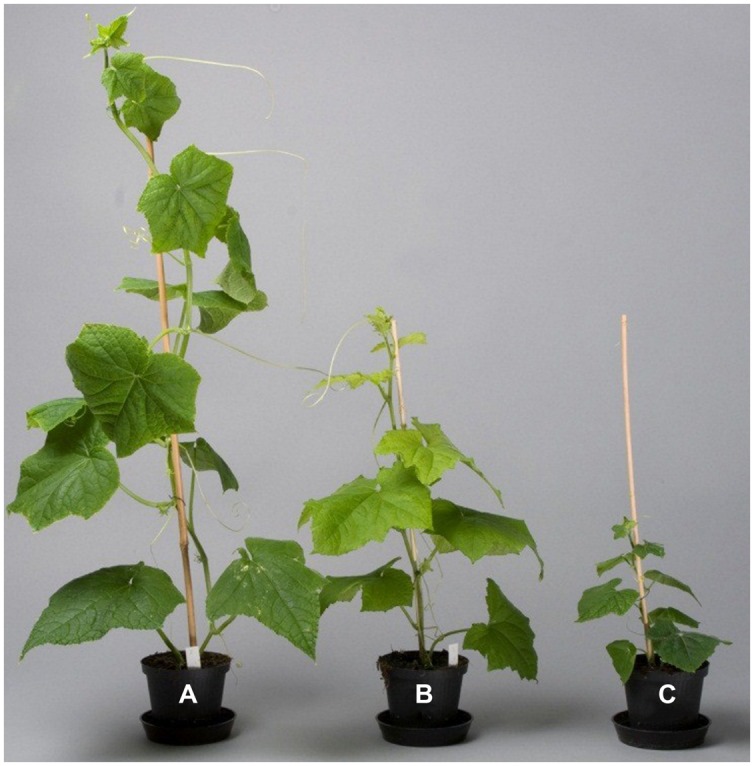
**Morphological difference of the three species: **(A)** ‘BejingJietou’; **(B)***Cucumis* × *hytivus*; and (**C)***Cucumis hystrix* photographed 3 weeks after propagation from cuttings grown under HL/SD**.

**FIGURE 2:**
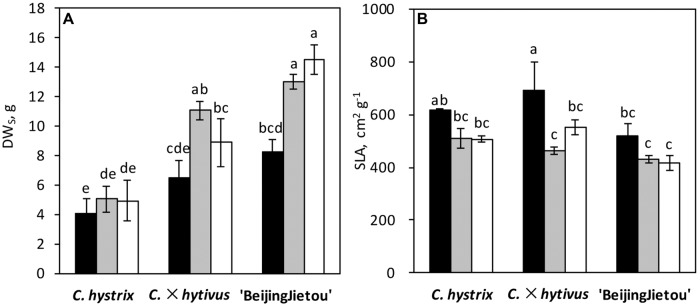
**Plant growth parameters: **(A)** shoot dry weight (DW_S_) and **(B)** specific leaf area (SLA) of three species after different light conditions for 21 days.** LL/SD, 70 μmol m^-2^ s^-1^ (black), IL/LD, 90 μmol m^-2^ s^-1^ (gray) and HL/SD, 140 μmol m^-2^ s^-1^ (white). Vertical bars represent the mean ± SE (*n* = 3). The different letters above the bars show significant difference at *P* < 0.05.

The three *Cucumis* species showed large differences in biomass production. *C. hystrix* (wild species) had a small plant size, whereas the hybrid *C.* × *hytivus* had medium size compared with the cultivated species ‘BejingJietou’ (**Figure 1**). After 21 days’ growth, *C. hystrix* had significantly smaller LA (1552 ± 422 cm^2^) than *C.* × *hytivus* (3058 ± 410 cm^2^) and ‘BejingJietou’ (3383 ± 165 cm^2^) under HL/SD (*P* < 0.05), whereas the LA of *C.* × *hytivus* and ‘BejingJietou’ was not significantly different despite the difference in plant height. Due to the absence of significant treatment effect on LA, data were pooled. Under HL/SD, significantly different DW_S_ were found between all three species, highest in ‘BejingJietou’ and lowest in *C. hystrix* (**Figure 2A**). Compared with HL/SD, the LL/SD treatment resulted in a decrease of DW_S_ in ‘BejingJietou,’ but not in *C. hystrix* and *C.* × *hytivus* (**Figure 2A**). Under LL/SD, the SLA increases compared with IL/LD and HL/SD, but significantly higher SLA was only observed in *C.* × *hytivus* (**Figure 2B**).

### Pigmentation

**FIGURE 3:**
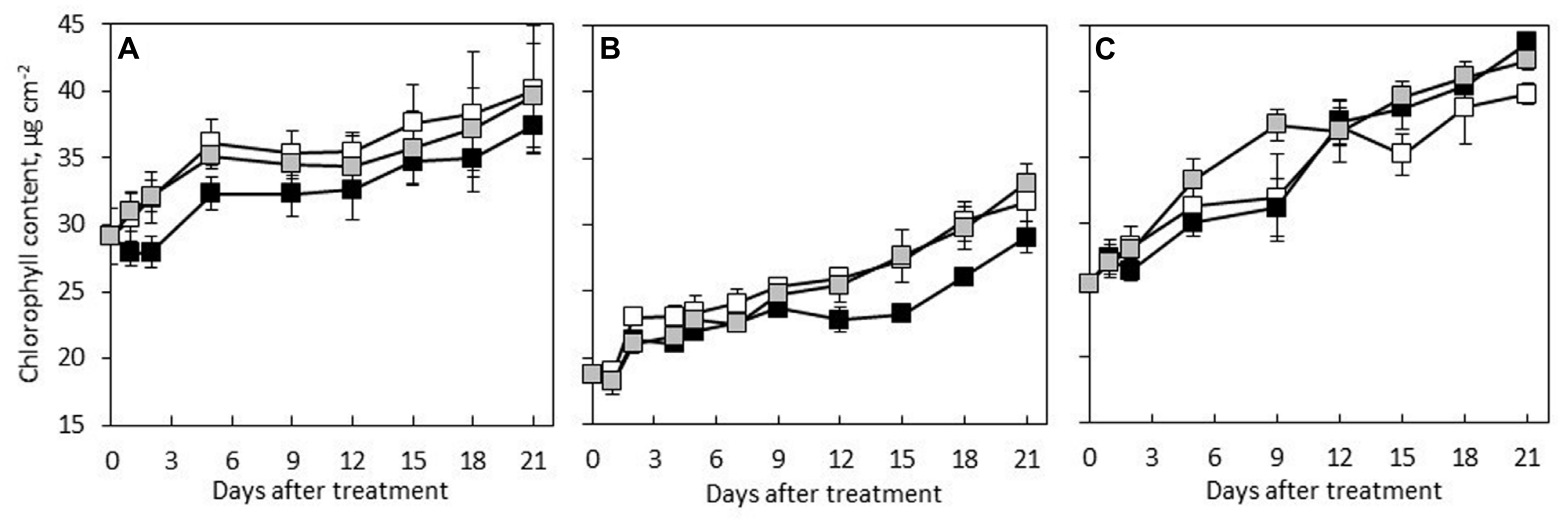
**Chl content of developing leaf in the three species measured by Dualex 4: **(A)***C. hystrix*; **(B)***C.* × *hytivus*; and (**C)** ‘BejingJietou’; LL/SD (black), IL/LD (gray) and HL/SD (white).** Vertical bars represent the mean values ± SE (*n* = 3).

The results showed that *C.* × *hytivus* had the lowest Chl content in young leaves among the three species under HL/SD, reflecting the light green color of young leaves, whereas the highest Chl content was observed in the dark green young leaves of *C. hystrix* (**Figure 3**). Regardless of the treatment, the three species followed a similar pattern showing increasing Chl content during the leaf development, though at varying rates. During the experimental period, the Chl content of *C. hystrix, C. × hytivus* and ‘BejingJietou’ increased by 38, 69, and 58%, respectively. Thus *C.* × *hytivus* had the largest increase in Chl content, reflecting the color change from yellow–green to ‘normal’ green. The non-invasive and destructive measurements of Chl *a* and Chl *b* content were significantly correlated (*R* = 0.581, *P* < 0.01 and *R* = 0.399, *P* < 0.05, respectively). Consistently, the extracted Chl content analysis showed species-dependent differences irrespective of light treatment (**Table 1**). However, the pigmentation of mature leaves of all the three species was not affected by the light treatments and therefore data were pooled. The significantly higher Chl *b* content observed in *C. hystrix* resulted in the highest total Chl content and a lower Chl *a*/*b* ratio of *C. hystrix* (**Table 1**). *C.* × *hytivus* had significantly lower Chl *a* than the parent species. Besides the difference in Chl, the mature leaves of *C.* × *hytivus* also had significantly lower Caro content (**Table 1**). Significantly higher Chl *a*/*b* ratio and lower Chl/ Caro ratio was observed in ‘BejingJietou.’

**Table 1:** Photosynthetic pigments Chl *a* and Chl *b*, total Chl, Chl *a/b* ratio, Caro and Chl/Caro ratio of the three *Cucumis* genotypes after 21 days.

Species	Chl *a* (mg g^-1^)	Chl *b* (mg g^-1^)	Total Chl (mg g^-1^)	Chl *a/b*	Caro (mg g^-1^)	Chl/Caro
*Cucumis hystrix*	54 ± 4.1^a^	35 ± 3.0^a^	89 ± 7.1^a^	1.5 ± 0.03^c^	7 ± 0.3^a^	13.1 ± 1.06^a^
*Cucumis* × *hytivus*	33 ± 2.4^b^	17 ± 1.4^b^	50 ± 3.8^b^	2.0 ± 0.05^b^	3 ± 0.4^b^	15.0 ± 1.25^a^
‘BejingJietou’	44 ± 3.4^a^	19 ± 1.6^b^	63 ± 5.0^b^	2.4 ± 0.05^a^	7 ± 0.5^a^	8.8 ± 0.37^b^

*Values represent mean ± SE (*n* = 3). Different letters in the same column show significant difference at 0.05 (*P* < 0.05).*

### Gas Exchange

**FIGURE 4:**
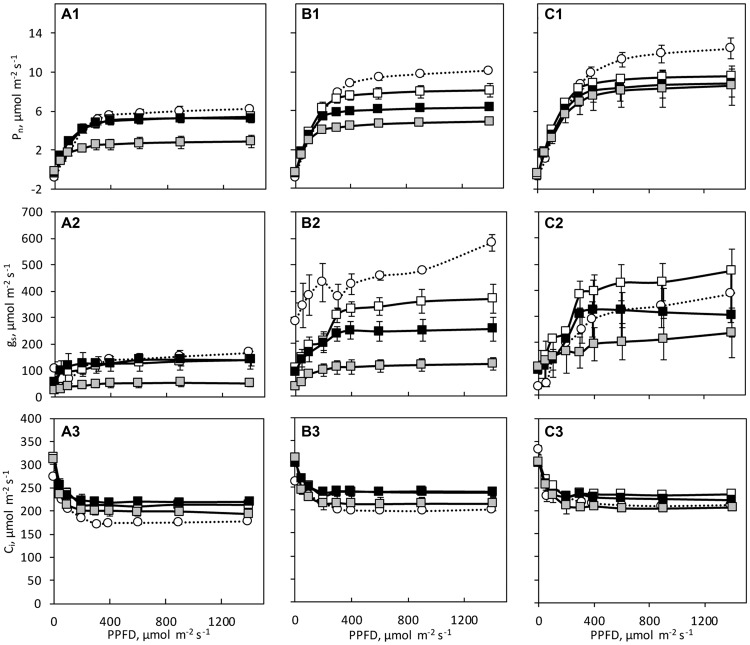
**Light response curves of the net photosynthesis (P_n_), stomatal conductance (g_s_), and internal CO_2_ concentration (C_i_) of **(A)***C. hystrix*; **(B)***C.* × *hytivus*; and **(C)** ‘BejingJietou’ before (day 0, circles, dashed lines) and after the light treatment (day 21, squares, solid lines); LL/SD (black), IL/LD (gray) and HL/SD (white).** The data are mean values ± SE (*n* = 3).

The three species showed different photosynthetic light response curves with significant species differences in LSP, *P*_max_, g_s_ and C_i_ at day 0 (**Figure 4** and **Table 2**). The *P*_max_ was lowest in *C. hystrix* and highest in ‘BejingJietou’ (**Table 2**). Significantly lower C_i_ was observed in *C. hystrix*, indicating a stomatal limitation (**Table 2**). Although *C.* × *hytivus* had significantly lower Chl content than its parents, the net photosynthesis rate (*P*_n_) was not negatively affected but showed intermediate values compared to the parents (**Figure 4**). The LSP in *C.* × *hytivus* and ‘BejingJietou’ were similar and significantly lower in *C. hystrix*.

**Table 2:** Photosynthetic characterization of the three species before treatment (Day 0) and after 2 and 21 days of treatment: dark respiration (*R*_d_), light compensation point (LCP), light saturating point (LSP), and maximum net photosynthesis rate (*P*_max_) measured at saturating light level.

	Species	Treatment	LSP (μmol m^-2^ s^-1^)	LCP (μmol m^-2^ s^-1^)	*R*_d_ (μmol m^-2^ s^-1^)	*P*_max_ (μmol m^-2^ s^-1^)
Day 0	*C. hystrix*		440 ± 74.1^b^	21.3 ± 8.9^a^	0.7 ± 0.2^a^	7.0 ± 0.3^c^
	*C. × hytivus*		695 ± 10.8^a^	17.7 ± 2.5^a^	0.8 ± 0.1^a^	11.0 ± 0.2^b^
	‘BejingJietou’		886 ± 73.3^a^	18.1 ± 7.0^a^	0.8 ± 0.3^a^	13.4 ± 1.5^a^
Day 2	*C. hystrix*	LL/SD	559 ± 56.7^cd^	17.8 ± 3.8^ab^	0.8 ± 0.1^ab^	6.7 ± 0.3^e^
		IL/LD	402 ± 26.4^d^	15.0 ± 0.9^bc^	0.4 ± 0.0^a^	4.1 ± 0.2^f^
		HL/SD	666 ± 15.3^bc^	25.0 ± 3.2^a^	1.1 ± 0.2^b^	8.2 ± 0.2^de^
	*C. × hytivus*	LL/SD	512 ± 81.3^cd^	11.2 ± 1.7^bcd^	0.5 ± 0.1^a^	9.2 ± 1.0^de^
		IL/LD	471 ± 59.3^cd^	13.1 ± 1.9^bc^	0.6 ± 0.1^a^	8.2 ± 0.5^de^
		HL/SD	652 ± 36.7^bcd^	10.3 ± 3.4^bc^	0.5 ± 0.2^a^	10.1 ± 0.8^cd^
	‘BejingJietou’	LL/SD	1039 ± 193.6^a^	6.6 ± 2.5^c^	0.4 ± 0.2^a^	13.7 ± 1.5^b^
		IL/LD	856 ± 28.5^ab^	7.5 ± 0.9^c^	0.4 ± 0.1^a^	12.3 ± 1.1^bc^
		HL/SD	955 ± 43.5^a^	10.3 ± 2.5^bc^	0.5 ± 0.1^a^	16.3 ± 0.8^a^
	Species effect		^∗∗^	^∗∗^	^∗^	^∗∗^
	Treatment effect		^∗^	–	–	^∗∗^
	Species × treatment effect		–	–	–	–
Day 21	*C. hystrix*	LL/SD	422 ± 14.2^ab^	6.5 ± 1.9^a^	0.3 ± 0.1^ab^	5.6 ± 0.5^cde^
		IL/LD	345 ± 64.5^b^	6.5 ± 0.9^a^	0.2 ± 0.0^a^	3.1 ± 0.6^e^
		HL/SD	459 ± 21.1^ab^	10.2 ± 0.9^a^	0.5 ± 0.0^bc^	5.9 ± 0.4^cd^
	*C. × hytivus*	LL/SD	376 ± 60.5^b^	8.4 ± 1.6^a^	0.4 ± 0.0^abc^	6.7 ± 0.1^bcd^
		IL/LD	395 ± 54.2^ab^	8.4 ± 1.6^a^	0.5 ± 0.1^bc^	5.3 ± 0.4^de^
		HL/SD	493 ± 24.6^ab^	8.4 ± 0.01^a^	0.5 ± 0.03^bc^	8.6 ± 0.66^abc^
	‘BejingJietou’	LL/SD	551 ± 51.4^ab^	7.5 ± 0.93^a^	0.4 ± 0.03^abc^	9.2 ± 1.42^ab^
		IL/LD	623 ± 152.3^a^	8.4 ± 1.63^a^	0.4 ± 0.11^abc^	9.1 ± 2.21^ab^
		HL/SD	573 ± 43.2^ab^	10.3 ± 1.87^a^	0.6 ± 0.11^c^	10.2 ± 0.36^a^
	Species effect		^∗^	–	^∗^	^∗∗^
	Treatment effect		–	–	^∗^	^∗^
	Species × treatment effect		–	–	–	–

*Values represent the mean ± SE (*n* = 3). Different letters in the same column show a significant difference at 0.05 (*P* < 0.05) within days of treatment. Level of significance (^∗^*P* < 0.05; ^∗∗^*P* < 0.01).*

After 2 days treatment of ‘BejingJietou’ no significant effect of LL/SD was detected on LSP, LCP and *R*_d_, whereas a significantly higher *P*_max_ was observed in HL/SD (**Table 2**). No effect of LL/SD was observed in any of the photosynthetic parameters of *C. hystrix* and *C.* × *hytivus* compared to HL/SD (**Table 2**).

We observed differences in the response of the three species to a long photoperiod in terms of *P*_max_. ‘BejingJietou’ displayed significantly lower *P*_max_ under IL/LD than under HL/LD only on day 2 (**Table 2**). The long photoperiod affected the *P*_n_ of *C. hystrix* differently from the effect of low PPFD. All the gas exchange parameters of *C. hystrix* under IL/LD were significantly lower under HL/SD on day 2. On day 21, there was a significant lower *P*_max_ and *R*_d_ in IL/LD (**Table 2**). There was no effect of IL/LD on *C.* × *hytivus*, but a significant decrease in *P*_max_ was observed on day 21 (**Table 2**).

### Chlorophyll Fluorescence

**FIGURE 5:**
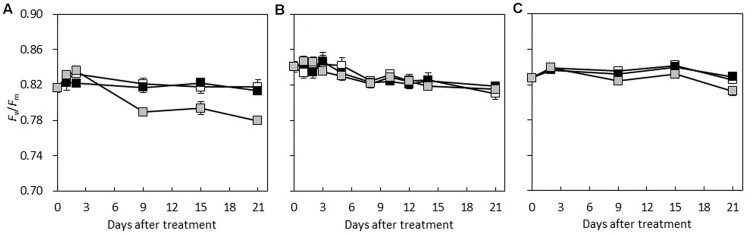
**The maximum quantum efficiency of PS II (*F*_v_/*F*_m_) of the first fully developed leaf of **(A)***C. hystrix*; **(B)***C.* × *hytivus*; and **(C)** ‘BejingJietou’ during the experimental period; LL/SD (black), IL/LD (gray) and HL/SD (white).** Vertical bars represent the mean values ± SE (*n* = 3).

**FIGURE 6:**
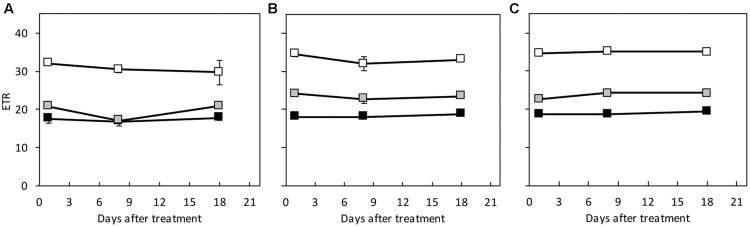
**The electron transport rate (ETR) measured on the first fully developed leaf of **(A)***C. hystrix*; **(B)***C.* × *hytivus*; and **(C)** ‘BejingJietou’ at respective mean light level; LL/SD, 70 μmol m^-2^ s^-1^ (black), IL/LD, 90 μmol m^-2^ s^-1^ (gray) and HL/SD, 140 μmol m^-2^ s^-1^ (white).** Vertical bars represent the mean values ± SE (*n* = 3).

Under HL/SD and LL/SD, no significant difference was observed in *F*_v_/*F*_m_ between the species (**Figure 5**). However, significant decrease in *F*_v_/*F*_m_ was observed in *C. hystrix* from day 9 of IL/LD treatment (**Figure 5**), indicating the PSII of *C. hystrix* could be impaired by the longer photoperiod. The ETR was compared statistically only within species because that the common leaf absorbance coefficient (0.84) for C_3_ plants (Björkman and Demmig, 1987; Krall and Edwards, 1992) were used for the calculation of ETR, which may be different among the three species due to their different Chl content (**Figure 6**). In *C.* × *hytivus* and ‘BejingJietou,’ ETR decreased in accordance to the light level. However, in *C. hystrix* after 8 and 18 days there was no significant difference between LL/SD and IL/LD. This may indicate that the wild species, *C. hystrix*, is actually more efficient in using the very low light of the LL treatment than the other two species (**Figure 6**).

## Discussion

In this study, we explored the response in three species of cucumber (*Cucumis*) toward changes in light level and photoperiod in terms of pigmentation and photosynthesis. *C.* × *hytivus* was obtained through an interspecific hybridization between two distinct diploid parents, *C. hystrix* and ‘BejingJietou,’ with an intention to broaden the cucumber genome base and to try to overcome the low light limitation of cucumber production. However, according to our results, the shade-tolerance is not passed on to *C.* × *hytivus*. Moreover, *C.* × *hytivus* showed Chl deficiency, which could be related to the interspecific cross.

Plants adapted to life in the bottom of a forest are excellent at exploiting low intensities of light and to do so, the light capturing part of the photosynthetic apparatus is optimized on the expense of the “dark” part of photosynthesis, i.e., the Calvin-Benson cycle (Boardman, 1977; Anderson, 1986; Skillman et al., 2005). Therefore shade-adapted plants have higher content of Chl per chloroplast combined with fewer chloroplasts per LA and a lower Chl *a/b* ratio (Anderson, 1986; Anderson et al., 1988; Terashima and Hikosaka, 1995). *C. hystrix* accumulates primarily Chl *b*, which is only found in the antenna systems, not in the core complex of the photosystems. It was often seen in shade plants (Boardman, 1977; Lichtenthaler et al., 2007; Baldi et al., 2012). Being originated from the bottom of rain forest (Chen et al., 1994), the shade-adaptation of *C. hystrix* is also proofed by the higher total Chl content and lower Chl *a/b* ratio. The differences in number and composition of Chl and chloroplasts in sun and shade plants/leaves may result in similar levels of Chl content per LA. The Chl *a*/*b* ratio can hereby be used as an indication of the balance in a plant between light harvesting and photosynthesis as this ratio correlates positively to the ratio of PSII core to LHCII (Anderson et al., 1988; Terashima and Hikosaka, 1995). When the Chl *a/b* ratio increased from *C. hystrix* to *C.* × *hytivus* and to the high light adapted ‘BeijingJietou,’ it indicates a decreasing size of LHCII. Although being lower in Chl *a*, the antenna size (Chl *a*/*b*) of *C.* × *hytivus* is intermediate to the parents. *C.* × *hytivus* is also low in Caro, but in combination with total Chl that is lower than the wild species, it can maintain as high Chl/Caro as the wild parent. ‘BeijingJietou’ is only different in smaller antenna (Chl *a*/*b*) and lower Chl/Caro than the others, i.e., features that protects against high light, which is advantageous for high light growing. In contrast, none of the Chl parameters showed significant changes induced by PPFD or photoperiod, suggesting these parameters to be genetically determined differences between the three species within the range of low PPFD used in this experiment.

To maximize light capturing in low light conditions, the light harvesting centers are often spread out in thin leaves instead of being organized in stacks (i.e., shade plants have higher SLA than sun plants). Under shade conditions, some species (e.g., *Fagus sylvatica, Raphanus sativus* L., *Triticum aestivum* L., *Zea mays, Tetrastigma* sp., and *Ginkgo biloba*) responded by an increase in SLA and photosynthetic pigments, especially Chl *b* (Lichtenthaler et al., 2007; Sarijeva et al., 2007; Dai et al., 2009). All three cucumber species had comparable SLA after the light treatments, meaning that there were no species specific acclimation potentials to the light treatments in respect to SLA. SLA can be affected by the thickness of leaves, amount of solutes and starch in the leaves. The cultivated ‘BeijingJietou,’ however, had slightly lower SLA compared to the other two species, in accordance with the hypothesis. The light treatments were at relatively low PFFD. Within that range only *C.* × *hytivus* responded with a significant difference to the lowest DLI of the LL/SD treatment.

It has long been known that species occupying shaded habitats have lower photosynthetic capacity (*P*_max_) and we also found consistently lower *P*_max_ in *C. hystrix* compared to the high light adapted ‘BeijingJietou’ before and after light treatments (Boardman, 1977). As a consequence of the low *P*_max_, shade plants are not capable of exploiting higher light levels over longer periods of time, as they are not able to upscale the downstream parts of photosynthesis and therefore the captured energy from light cannot be transferred onward due to low levels of Calvin-Benson cycle enzymes, e.g., ribulose-1,5-bisphosphate carboxylase/oxygenase (Rubisco; Lichtenthaler et al., 2007). This, combined with low respiration (*R*_d_), which is also often found in shade species (Lichtenthaler et al., 1981; Wittmann et al., 2001), leads to lower growth rates and low biomass production (Skillman et al., 2005). Tognetti (1997) stated that under low light condition, the low light tolerant plants would still be able to maintain a relatively high photosynthetic rate, as seen in *C. hystrix*. In this species, the total amount of net carbon fixation has been similar in all light treatments. Together with the unchanged ETR between and LL/SD and IL/LD, *C. hystrix* is demonstrated low-light adapted for efficient use of light under low light conditions. Contrary, ‘BeijingJietou’ exhibited significantly reduced DW_S_ under LL/SD compared to that under IL/LD and HL/SD, which related to DLI. Also, *C.* × *hytivus* appears to some extent to be inhibited by the low light levels similarly to the cultivated ‘BeijingJietou,’ which suggests the low-light tolerance of *C. hystrix* is not passed on to *C.* × *hytivus*, who share more similarity with its’ father-plant, ‘BeijingJietou.’ However, it can be turned around to something positive that *C.* × *hytivus* and ‘BeijingJietou’ were able to utilize the extra light given in IL/LD and HL/SD, even though the increase in PPFD only was from 70 to 90 and 140 μmol m^-2^ s^-1^, which is practically possible to maintain in the winter production of cucumber in modern greenhouse, whereas *C. hystrix* cannot. Therefore the results do not support the hypothesis of heterosis or dosage effect advantage in *C.* × *hytivus* as this species does not seem to be able to tolerate and acclimate to low light in a higher degree than its parental species.

When comparing at a species level, the highest levels of *P*_max_ and DW_S_ were generally found in ‘BejingJietou,’ intermediate in *C.* × *hytivus* and lowest in *C. hystrix*. Both the stomatal and non-stomatal components contribute to the difference in photosynthetic rate (Quick et al., 1992). The value of the internal CO_2_ concentration (C_i_) can be used to distinguish between these two components (Farquhar and Sharkey, 1982). Significantly lower C_i_ was observed in *C. hystrix*, indicating that the lower *P*_max_ could be partly caused by stomata limitation. This is supported by the level of g_s_ being less than half of the rates in ‘BeijingJietou’ thus lower than for the hybrid *C.* × *hytivus* (**Table 2**). All these add together to result in the difference of *P*_n_ and contributing to their difference in biomass.

### Chl Deficiency

Chl deficiency induced by interspecific hybridization has been widely reported in *Medicago* (Lesins, 1961), *Melilotus* (Sano and Kita, 1978), *Brassica* (Prakash and Chopra, 1988; Chang et al., 2014), *Zantedeschia* (Yao et al., 1994), and *Gossypium* (Zhang et al., 2014). This is also the case with *C.* × *hytivus*, which had the lowest Chl content, in accordance with the light green color, in particular of young leaves. Interspecific segregants with Chl deficiency often suffer from lethal or reduced fitness due to the reduced photosynthesis (Zhang et al., 2014). However, although *C.* × *hytivus* showed lower Chl content in the young leaves the species was not adversely affected in photosynthesis. Moreover, the Chl content increased in all three species during the treatment period, where *C. × hytivus* had the highest increase, thereby approaching ‘normal’ green level. The *C.* × *hytivus* was intermediate between the parent species in most photosynthetic parameters.

### *C. × hytivus* – not Always an Intermediate

Looking particularly at the levels of Chl and Caro, it becomes obvious that *C.* × *hytivus* is not an intermediate hybrid with respect to these parameters. The lack of difference in *F*_v_/*F*_m_ between the species indicates that PSII was fully functional also in *C.* × *hytivus*, despite its lower content of Chl. Also, *C.* × *hytivus* did not show an intermediate response in SLA compared to its parents. The reduced Chl content could be the result of genomic shock and/or plastome–genome incompatibility. When two genomes are combined into one cell, some species have responded to the consequences of duplicate copies of genes, a phenomenon called “genomic shock” proposed by McClintock (1984). In this situation novel phenotypes arise that lack or differ from the features of the contributing parents, which cannot be explained by classical genetic rules (Kashkush et al., 2012). This could also be the explanation for the low level of Caro seen in *C.* × *hytivus*, compared to the parental species. The Caro are not only essential components of the photosynthetic antenna and reaction center complexes, but also protect the leaves against potentially harmful photo-oxidative processes, when the light energy exceeds the photosynthetic capacity (Bartley and Scolnik, 1995). Hence, the lower Caro content of *C.* × *hytivus* could indicate reduced tolerance to high light. A likewise combination of reduced content of Caro and Chl was also reported in the interspecific hybridization between *Brassica rapa* and *B. juncea* (Chang et al., 2014).

### Acclimatization Potential

Besides the genetically determined adaptation of the plants toward sun or shade, most plants are able to acclimatize to changes in the light level on short or long term (Ball and Critchley, 1982; Bailey et al., 2004; Baldi et al., 2012). How plants acclimatize efficiently is determined by the plasticity in response to the change in the surroundings, e.g., a change in PPFD (Sultan, 2000). Though none of our species showed acclimation of the Chl content to the light treatments, the light response curves before and after the treatments showed differences in acclimatization potential. All light treatments resulted in lower *P*_max_ in both *C*. × *hytivus* and ‘BeijingJietou,’ which could be due to the higher PPFD in the greenhouse before the start of the treatment, though only *C*. × *hytivus* showed significantly different light response curves in the different treatments. There were no indications on severe stress imposed by the PPFD, as the *F*_v_/*F*_m_ values were not changed. However, the long photoperiod affected the shade adapted *C. hystrix*. With time the IL/LD treatment resulted in lower *F*_v_/*F*_m_ indicating that the PSII of *C. hystrix* was mildly impaired by the longer photoperiod, combined with lower g_s_ resulting in lower *P*_max_. Interestingly, the *F*_v_/*F*_m_ in *C. hystrix* was not affected in the first 3 days of IL/LD treatment, suggesting that the prolonged photoperiod did not impair the PSII on the short term but developed over time. However, this effect of long photoperiod was not observed in *C.* × *hytivus*. Therefore, it is clear from the results that the sensitivity toward day length has not been passed on to the new species, *C.* × *hytivus*. The placement of the IL/LD as the lowest curve may point to some degree of inheritance of the inhibiting effect of long photoperiod on the wild parent, *C. hystrix*, as it only showed a significant acclimation of the light response when grown at the IL/LD treatment.

The genus *Cucumis* was initially divided into two subgenera, *Melo* and *Cucumis*. While the subgenus *Melo* is centered in Africa with 30 species including melon (all of which have 12 chromosome pairs), the subgenus *Cucumis* is of Asian origin and includes the cultivated cucumber *C. sativus* (seven chromosome pairs) and its wild relative *C. hystrix* (12 chromosome pairs). The successful cross represents a breakthrough in interspecific hybridization in *Cucumis*. The restoration of fertility marked the creation of a new synthetic species, *C*. × *hytivus*, which has close phylogenetic relationships with its parental species, but is distinctively different from them (Chen and Kirkbride, 2000). That makes it an excellent model to study the interspecific hybridization effect. Although the results showed that the assumed shade tolerance was not passed onto *C.* × *hytivus*, the research presented here is very valuable as the first photosynthetic characterization of this new species and an example of investigating the physiological effect of allopolyploidization. Moreover, *C. × hytivus* contains the whole genome of the wide germplasm, which means that it can be further used as a bridging material to create introgression lines that contains fragment or genes of *C. hystrix* genome for cucumber improvement, including shade tolerance, an example is the increased disease resistance of the introgression lines (Wan et al., 2010).

## Conclusion

The results show that the new *C. × hytivus* only partly is an intermediate hybrid and that it recovers from Chl deficiency during leaf development. This makes this hybrid an interesting and relevant model to study the mechanisms of genomic shock and plastome–genome interaction in allopolyploidization. The expected ability of *C. × hytivus* to tolerate low light conditions was not improved compared to the capacity of the parents.

## Conflict of Interest Statement

The Associate Editor Soren K. Rasmussen declares that, despite being affiliated with the same institute as the author Eva Rosenqvist, the review process was handled objectively. The authors declare that the research was conducted in the absence of any commercial or financial relationships that could be construed as a potential conflict of interest.
